# Supplementary material for: Phenotypic and Molecular Characteristics of the MDR Efflux Pump Gene-Carrying Stenotrophomonas maltophilia Strains Isolated in Warsaw, Poland
Source: Biology (Basel). 2022 Jan 10;11(1):105. doi: 10.3390/biology11010105 (PMC8772754; doi:10.3390/biology11010105)
Supplement: Supplementary file 1 [file biology-11-00105-s001.zip › biology-1520413-supplementary.pdf]

Supplementary material.

Table S1. Presence of efflux pump genes in *S. maltophilia* isolates (*n*=94).

No	Isolates	*smeD* (*smeD*)[a]	*smeB* (*smeA*)[a]	*smeK* (*smeI*)[a]	*smeZ*	*smeW* (*smeV*)[a]	*smeH*	*smeP*	*smeN*	*smrA*	*macB*
1	1/2010	+	+	+	+	+	+	-	+	+	+
2	2/2010	+	- (-)	+	+	+	+	+	+	+	+
3	3/2010	+	- (+)	+	+	+	+	+	+	+	+
4	4/2010	+	+	+	+	+	+	-	+	+	+
5	5/2010	+	- (-)	- (+)	+	+	+	+	+	+	+
6	6/2010	+	+	+	+	+	+	+	+	+	+
7	7/2010	+	+	+	+	+	+	+	+	+	+
8	8/2010	+	+	+	+	+	+	+	+	+	+
9	9/2010	+	+	+	+	+	+	-	+	+	+
10	10/2010	+	+	+	+	+	+	+	+	+	+
11	11/2010	+	+	+	+	+	+	+	+	+	+
12	12/2010	+	+	- (+)	+	+	+	+	+	+	+
13	13/2010	+	+	- (-)	+	+	+	+	+	+	+
14	14/2010	+	+	+	+	+	+	+	+	+	+
15	15/2010	+	+	- (+)	+	+	+	-	+	+	+
16	16/2010	+	+	- (+)	+	+	+	+	+	+	+
17	17/2010	+	+	- (+)	+	+	+	+	+	+	+
18	18/2010	+	+	+	+	+	+	+	+	+	+
19	19/2010	+	+	+	+	+	+	+	+	+	+
20	20/2011	- (+)	- (-)	+	+	+	+	+	+	-	+
21	21/2011	+	- (-)	+	+	+	+	+	+	+	+
22	22/2011	+	+	+	+	+	+	+	+	+	+
23	23/2011	+	+	+	+	+	+	+	+	+	+
24	24/2011	+	+	- (+)	+	+	+	+	+	+	+
25	25/2011	+	+	- (+)	+	+	+	+	+	+	+
26	26/2011	+	+	- (+)	+	+	+	+	+	+	+
27	27/2011	+	+	- (+)	+	+	+	+	+	+	+
28	28/2011	+	+	+	+	+	+	+	+	+	+
29	29/2011	+	+	+	+	+	+	+	+	+	+
30	30/2011	+	+	+	+	+	+	+	+	+	+
31	31/2011	+	+	+	+	+	+	+	+	+	+
32	32/2011	- (+)	- (-)	+	+	+	+	+	+	+	+
33	33/2011	+	- (-)	- (-)	+	+	+	+	+	+	+
34	34/2011	+	+	- (+)	+	+	+	+	+	+	+
35	35/2011	+	- (-)	- (-)	+	+	+	+	+	+	+
36	36/2011	+	- (-)	- (-)	+	+	+	+	+	+	+
37	37/2011	+	+	+	+	+	+	+	+	+	+
38	38/2011	+	+	+	+	+	+	+	+	+	+
39	39/2011	- (+)	- (-)	+	+	+	+	+	+	+	+
40	41/2011	+	+	+	+	+	+	+	+	+	+
41	42/2011	+	- (-)	+	-	+	+	+	+	+	+
42	43/2011	+	+	+	+	+	+	+	+	+	+
43	44/2011	+	- (-)	+	+	+	+	+	+	+	+

44	45/2011	+	+	+	+	+	+	+	+	+	+
45	46/2011	+	+	+	+	+	+	+	+	+	+
46	47/2011	+	- (-)	+	+	+	+	+	+	+	+
47	48/2011	+	+	+	+	+	+	+	+	+	+
48	49/2011	+	+	+	+	+	+	+	+	+	+
49	50/2012	+	+	+	+	+	+	+	+	+	+
50	51/2012	+	+	+	-	+	+	+	+	+	+
51	52/2012	- (+)	- (+)	+	+	+	+	+	+	+	+
52	53/2012	+	+	+	+	+	+	+	+	+	+
53	54/2012	+	+	+	-	+	+	+	+	+	+
54	55/2012	+	+	- (-)	+	+	+	+	+	+	+
55	56/2012	+	+	- (+)	+	+	+	+	+	+	+
56	57/2012	+	+	+	+	+	+	+	+	+	+
57	58/2012	+	+	+	+	+	+	+	+	+	+
58	59/2012	+	+	- (+)	+	+	+	+	+	+	+
59	60/2012	+	+	+	+	+	+	+	+	+	+
60	61/2012	+	+	- (+)	+	+	+	+	+	+	+
61	62/2012	+	+	- (+)	+	+	+	+	+	+	+
62	63/2012	+	+	- (+)	+	+	+	+	+	+	+
63	64/2012	+	+	+	+	+	+	+	+	+	+
64	65/2012	+	+	+	+	+	+	+	+	+	+
65	66/2013	+	- (-)	+	+	+	+	+	+	+	+
66	67/2013	+	+	+	+	+	+	+	+	+	+
67	68/2013	+	+	+	+	+	+	+	+	+	+
68	69/2013	+	+	- (+)	+	+	+	+	+	+	+
69	70/2013	- (+)	- (-)	+	+	+	+	+	+	+	+
70	71/2013	+	+	+	+	+	+	+	+	+	+
71	72/2013	+	- (-)	+	+	+	+	+	+	+	+
72	73/2013	+	+	+	+	+	+	+	+	+	+
73	74/2013	+	+	+	+	+	+	+	+	+	+
74	75/2013	+	+	+	+	- (-)	+	+	+	+	+
75	76/2013	+	+	+	+	+	+	+	+	+	+
76	77a/2013	+	+	+	+	+	+	+	+	+	+
77	77b/2013	+	+	+	+	+	+	+	+	+	+
78	78a/2013	+	+	+	+	+	+	+	+	+	+
79	78b/2013	+	+	+	+	+	+	+	+	+	+
80	79/2013	+	+	+	+	+	+	+	+	+	+
81	80/2013	+	+	+	+	+	+	+	+	+	+
82	81/2013	+	+	+	+	+	+	+	+	+	+
83	82/2013	+	- (-)	+	+	+	+	+	+	+	+
84	83/2013	+	+	+	+	+	+	+	+	+	+
85	84/2013	+	- (-)	+	+	+	+	+	+	+	+
86	85/2011	+	+	+	+	+	+	+	+	+	+
87	88/2011	+	- (+)	- (+)	+	+	+	+	+	+	+
88	89/2011	+	- (-)	+	-	+	+	+	+	+	+
89	90/2011	+	- (-)	- (-)	+	+	+	+	+	+	+
90	91/2013	+	+	+	+	+	+	+	+	+	+
91	92/2013	+	- (-)	- (-)	+	+	+	+	+	+	+
92	93/2013	+	+	+	+	+	+	+	+	+	+

93	94/2013	+	- (-)	+	-	+	+	+	+	+	+
94	95/2013	+	- (-)	- (-)	+	+	+	+	+	+	+

+, positive result of gene amplification; -, negative result of gene amplification.
[a]For negative results of efflux pump gene amplification, the reaction was repeated with new primers (results in parentheses).
